# Supplementary material for: Elevated Thyroxine Concentration and Lithium Intoxication—An Analysis Based on the LiSIE Retrospective Cohort Study
Source: J Clin Med. 2022 May 27;11(11):3041. doi: 10.3390/jcm11113041 (PMC9181082; doi:10.3390/jcm11113041)
Supplement: Supplementary file 1 [file jcm-11-03041-s001.zip › jcm-1724373-supplementary.pdf]

Journal of
Clinical Medicine

MDPI

Article

Elevated Thyroxine Concentration and Lithium Intoxication—An Analysis Based on the LiSIE Retrospective Cohort Study

Ingrid Lieber [1,*], Michael Ott [2], Robert Lundqvist [3], Mats Eliasson [3], Mikael Sandlund [4] and Ursula Werneke

[1] Department of Clinical Sciences, Psychiatry, Sunderby Research Unit, Umeå University, 90185 Umeå, Sweden; Ingrid.lieber@umu.se
[2] Department of Public Health and Clinical Medicine, Umeå University, 90185 Umeå, Sweden.
[3] Department of Public Health and Clinical Medicine, Sunderby Research Unit, Umeå University, 90185 Umeå, Sweden.
[4] Division of Psychiatry, Department of Clinical Sciences, Umeå University, 90185 Umeå, Sweden.
* Correspondence: Ingrid.Lieber@umu.se

Citation: Lieber, I.; Ott, M.; Lundqvist, R.; Eliasson, M.; Sandlund, M. Elevated Thyroxine Concentration and Lithium Intoxication—An Analysis Based on the LiSIE Retrospective Cohort Study. *J. Clin. Med.* **2022**, *11*, 3041. https://doi.org/10.3390/jcm11113041

Academic Editor(s): Rasa Zarnegar

Received: 27 April 2022
Accepted: 25 May 2022
Published: 27 May 2022

Publisher's Note: MDPI stays neutral with regard to jurisdictional claims in published maps and institutional affiliations.

Copyright: © 2022 by the authors. Submitted for possible open access publication under the terms and conditions of the Creative Commons Attribution (CC BY) license (https://creativecommons.org/licenses/by/4.0/).

Table S1: STROBE Statement - checklist for our study

STROBE requirement	#	Our study
Title and abstract	1	
(a) Indicate the study's design with a commonly used term in the title and abstract		(a) Given: "Elevated thyroxine concentration and lithium intoxication – an analysis based on the LiSIE retrospective cohort study"
(b) Provide in the abstract an informative and balanced summary of what was done and what was found		(b) Structured abstract provided.
Introduction		
Background/rationale: Explain the scientific background and rationale for the investigations being reported	2	Background outlined in introduction.
Objectives: State specific objectives, including any pre-specified hypotheses	3	Aims clearly stated in text, "We sought to determine the relevance of hyperthyroxinaemia as a risk factor for lithium intoxication. Specifically, we tested the following hypotheses:<br>1. Hyperthyroxinaemia is commonly associated with lithium intoxication.<br>2. Hyperthyroxinaemia leads to increased tubular reabsorption of lithium, which increases the risk of lithium intoxication."
Methods		
Study design: Present key elements of the study design early in the paper	4	Study design: we performed an analysis based on the LiSIE retrospective cohort study. Key elements of the study included in the manuscript: study design, participants, selection: inclusion and exclusion criteria, outcome definition, exposure parameters, variable definitions, validation process, control for bias, missing data and statistical analysis.
Setting: Describe the setting, locations, and relevant dates, including periods of recruitment, exposure, follow-up, and data collection	5	Setting and all relevant dates described in manuscript: "LiSIE invited all individuals in the Swedish regions of Västerbotten and Norrbotten ≥18 years of age, who had (a) received a diagnosis of bipolar disorder (BD) (ICD10 F31) or schizoaffective disorder (SZD) (ICD10 F25), or (b) used lithium as a mood stabiliser between 1997 and 2011"<br>"For the current study, we considered patients from the region of Norrbotten who had received a diagnosis of either BD or SZD on at least two occasions, at least six months apart at any time between 1997 and 2013. We then selected all patients who had been treated with lithium at some time during a 21-year review period from 1997 to 2017. For patients with lithium exposure, we identified patients who had experienced at least one episode of lithium intoxication"
Participants: *(a)* Give the eligibility criteria, and the sources and methods of case ascertainment and control selection. Give the rationale for the choice of cases and controls *(b)* For matched studies, give matching criteria and	6	(a) As above<br>"For the outcomes and exposure variables, we retrospectively reviewed the medical records of all eligible patients from 1997 up to 31st December 2017. From the medical records, we manually validated the date of the electronic prescriptions when lithium had been started or discontinued."<br><br>(b) N/A.

the number of controls per case		
Variables:<br>Clearly define all outcomes, exposures, predictors, potential confounders, and effect modifiers. Give diagnostic criteria, if applicable	7	Definition for exposures and variables given in text. "The outcome for this study was episodes of unintentional lithium intoxication. Here we considered only clinically relevant intoxications with a lithium serum concentration (s-lithium) of at least 1.5 mmol/L."<br>Lithium exposure: "Proof of lithium exposure was determined by at least one blood lithium concentration >0.2 mmol/L. As we investigated a potential adverse effect of lithium treatment but not therapeutic effect, we did not require lithium concentrations to be therapeutic. For each patient, we validated lithium start and stop date using available lithium concentrations, electronic prescriptions, and information from the medical records. Observed time in the study was measured from 1st of January 1997 to 31st of December 2017. The observation time for each individual patient started at the date of lithium initiation. For patients who moved out of the region or died before 31st of December 2017, the observation time stopped at the date of their departure or death. We estimated the time of lithium exposure in person-years."<br>Hyperthyroxinaemia: "We considered patients to have experienced an episode of hyperthyroxinaemia if they had fT4 above the upper reference range. We only considered episodes of hyperthyroxinaemia that had occurred after initiation of lithium. Most tests were analysed with an immunoassay from Roche Diagnostics Scandinavia with normal range reference values for thyroid function tests of 12.0 – 22.0 pmol/L for fT4. Hyperthyroxinaemia at the time of lithium intoxication was determined by fT4 tests taken within three months prior the intoxication. If several tests were available, we chose the test closest before the lithium intoxication."<br>Renal function: "We explored renal function before and during lithium intoxication. Creatinine in serum samples had been measured using an assay traceable to isotope dilution mass spectrometry (IDMS) creatinine. From creatinine, we calculated the estimated glomerular filtration rate (eGFR) using the CKD-EPI formula."<br><br>We also recorded age, sex, and type of underlying disorder. For episodes of lithium intoxication, we explored co-medications at time of lithium intoxication that might have interfered with the renal clearance of lithium.
Data sources /measurement:<br>For each variable of interest, give sources of data and details of methods of assessment (measurement). Describe comparability of assessment methods if	8	Data source for all variables: electronic medical records.<br><br>Definition for each variable given in text.

there is more than one group		
Bias:<br>Describe any efforts to address potential sources of bias	9	Potential sources of bias discussed, including selection and observer bias.<br>"We controlled for selection bias in the whole retrospective cohort study (LiSIE) using key parameters available in anonymised form. These included age, sex, and where applicable, maximum recorded lithium and creatinine concentrations. In accordance with the ethics approval granted, we compared these parameters for consenting and non-consenting patients. No significant difference was found between the two groups."
Study Size:<br>Explain how the study size was arrived at	10	Cf. figure 1<br>"1562 patients were included in the study(…). Of these, 897 patients had been exposed to lithium at any time during the review between 1997 and 2017"
Quantitative variables:<br>Explain how quantitative variables were handled in the analyses. If applicable, describe which groupings were chosen and why	11	Main outcome: Outcome summarized in the following categories:<br>(1) Incidence of unintentional lithium intoxication per 1000 person-years,<br>(2) Incidence of hyperthyroxinaemia per 1000 person-years,<br>(3) Incidence of hyperthyroxinaemia diagnosed at the time of lithium intoxication per 1000 person-years.
Statistical methods:<br>*a)* Describe all statistical methods, including those used to control for confounding<br>*(b)* Describe any methods used to examine subgroups and interactions<br>(*c)* Explain how missing data were addressed<br>*(d)* If applicable, explain how matching of cases and controls was addressed<br>*(e)* Describe any sensitivity analyses	12	(a) For hypothesis 1, we calculated the observed incidence of hyperthyroxinaemia-associated unintentional lithium intoxication based on identified episodes. We expressed the incidence in episodes/ person years. For hypothesis 2, we qualitatively explored the observed episodes of hyperthyroxinaemia-associated unintentional lithium intoxication.<br>(b) We examined if any unintentional lithium intoxication identified might be explained by increased tubular reabsorption attributable to hyperthyroxinaemia, or whether there was an alternative, more plausible, explanation. This could be GFR impairment due to dehydration or comedication with medicines affecting renal function.<br>(c) The data was complete for the defined outcome because lithium intoxication tended to be well documented and followed up. For one episode, a pre-intoxication fT4 measurement was not available.<br>(d) N/A<br>(e) N/A
Results		
Participants:<br>*(a)* Report numbers of individuals at each stage of study—eg numbers potentially eligible, examined for eligibility, confirmed eligible, included in the study, completing follow-up, and analyzed<br>*(b)* Give reasons for non-participation at each stage<br>*(c)* Consider use of a flow diagram	13	(a+b) Of 1562 included patients with BD or SZD, A total of 897 (57.4%) patients met our inclusion criteria (Figure 1).<br>(c) Flow diagram included in the manuscript as figure 1.
Descriptive data:	14	(a) Baseline characteristics described in table 2 of the manuscript.

(a) Give characteristics of study participants (e.g. demographic, clinical, social) and information on exposures and potential confounders<br>*(b)* Indicate number of participants with missing data for each variable of interest		(b) Included in the flow diagram and in the text.
Outcome data:<br>Report numbers in each exposure category, or summary measures of exposure	15	Outcome data presented in text and in table 3.
Main results<br>*(a)* Give unadjusted estimates and, if applicable, confounder-adjusted estimates and their precision (eg, 95% confidence interval). Make clear which confounders were adjusted for and why they were included<br>*(b)* Report category boundaries when continuous variables were categorized<br>*(c)* If relevant, consider translating estimates of relative risk into absolute risk for a meaningful time period	16	(a) Results presented according to the statistical method outlined in item 12<br>(b) Results presented according to the statistical method outlined in item 12. Variable definitions given in method.<br>(c) N/A
Other analysis:<br>Report other analyses done—e.g. analyses of subgroups and interactions, and sensitivity analyses	17	Sub-analysis according to the two hypotheses, cf. item 13
Discussion		
Key results:<br>Summarize key results with reference to study objectives	18	Done
Limitations:<br>Discuss limitations of the study, taking into account sources of potential bias or imprecision. Discuss both direction and magnitude of any potential bias	19	Limitation discussed in regard to selection bias, data quality, and potential for observer bias/recording error.

Interpretation: Give a cautious overall interpretation of results considering objectives, limitations, multiplicity of analyses, results from similar studies, and other relevant evidence	20	Results discussed in view of the limitations (weaknesses) of our study design. Advantages and disadvantages of studies based on medical records compared to register studies discussed.
Generalisability: Discuss the generalizability (external validity) of the study results	21	Discussed in the context of bias. The sample under study is judged to be representative and the largest sample available for the topic under study.
Funding: Give the source of funding and the role of the funders for the present study and, if applicable, for the original study on which the present article is based	22	This work was supported by a grant of the Research & Development Fund of Norrbotten Region, Sweden.<br><br>Conflict to interest statement for all authors included in manuscript.

Source: http://www.strobe-statement.org/. Accessed 12 March 2019.
